# Chicago Academy of Medical Sciences

**Published:** 1860-02

**Authors:** Delaskie Miller

**Affiliations:** President


### CHICAGO ACADEMY OF MEDICAL SCIENCES.

—

DELASKIE MILLER, M. D., PRESIDENT.

—

The regular monthly meeting was held on Friday, Jan. 6th, when the following officers were elected to serve during the ensuing year:

*President*—Dr. R. C. Hammill. *Vice President*—Dr. I. A. Graham. *Recording Secretary*—Dr. Walter Hay. *Corresponding Secretary*—Dr. John H. Rauch. *Treasurer*—Dr. Thomas Bevan. *Trustee*—Dr. Chas. G. Smith.

The following Standing Committees were also chosen;

*On Admissions*—Drs. Heydock, McAllister, and Holmes.

" *Medical Ethics*—Drs. Bloodgood, Fisher, and O. Smith.

" " *Education*—Drs. Davis, Ingals, and Gore.

A vote of thanks was given to the retiring officers.

Dr. Bloodgood (from Board of Trustees) reported that they had found suitable rooms for the permanent location of the Academy, and were thereupon authorized to secure such rooms as in there discretion might seem best, at a rent not to exceed $150 per annum.

Dr. Rauch moved that the Academy subscribe for the principal Medical Periodicals.

Dr. Byford moved an amendment to the effect that publishers be requested to donate, which was passed.

Dr. Rauch suggested the necessity of Incorporation in order to effect this object, whereupon the Secretary was instructed to take the necessary steps to effect the Incorporation of the Academy under the general Law of Incorporation.

Dr. Blake proposed the following resolution: *Resolved*, That Tracheomety is usually deferred too long in cases of membranous Croup, and that if resorted to earlier it would greatly lessen the mortality in that disease; which was accepted, and Drs. Bloodgood and Blake were appointed to discuss the subject at the next regular meeting.

Dr. McAllister called attention to a verdict of a Coroner's Jury, which had come under his notice, "That the subject died from disease of the stomach," no post-mortem examination having been made to determine the fact.

Dr. Gore (County Physician) had seen the case, and stated that no post-mortem examination was permitted by the parents of the boy; that the "verdict" in question was made up from the opinions of two physicians, who were said to be acquainted with the facts of the case, and from marks of violence upon the body, viz: an abrasion of the skin, and was that "The boy came to his death from internal injuries, (probably hæmorrhage) the results of a fall upon the edge of a portion of a barrel, while engaged in feeding cows." He also remarked upon the unreliable character of reports of such verdicts as published in newspapers, and assigned the reasons.

Dr. McAllister thought that juries were not justified in giving such verdicts without having post-mortem examinations.

Dr. Davis denied the right of parents, or any one else, to interfere with the rights of juries, maintaining that the subject of such an investigation belonged to the jury alone.

The subject was discussed at large by Drs. Davis, Blake, Gore, Ingals, and McAllister.

Dr. C. G. Smith suggested the propriety of exerting the influence of the Academy as a body in procuring the election of a physician to the office of Coroner.

Drs. Davis and Ingals were appointed a committee to investigate the subject of abuse in the practices of Coroner's Juries.

Dr. Bloodgood submitted the following report of the section to which the subject had been referred, viz:

The signs usually indicating death by drowning are the following: When found soon after death the surface of the body is pale or slightly livid, with muscular rigidity more or less complete, according to the time that has passed since the immersion took place; the eyes are partly open, and the pupils dilated the tongue is pressed against the teeth, or protruded between them, and sometimes wounded by them. The ends of the fingers are often abraded by the efforts made in the death struggle; the hands inclose grass or other substances that come within their reach, and the hollows of the nails are filled with sand or mud that forms the bed of the water. The abdomen and chest are prominent. The lungs and right side of the heart are filled with black fluid blood, as are also the liver and spleen, and a small quantity of bloody urine is sometimes found in the bladder. The stomach contains water of the same nature as that in which the body is found, and the air passages are more or less filled with froth formed by the water drawn in by the inspiratory efforts, and mingled with the air already present. The air found in the lungs is almost wholly destitute of oxygen. According to Prof. Beatty there is turgesence of the blood vessels in the head, more or less complete as the death has been more or less speedy; but Dr. Goodwin, as quoted by Roget, says that the external surface of the brain is of a darker color than usual, but the vessels are not tinged with blood, nor are there any marks of extravasation about them. Dr. Currie also says, they do not exhibit any particular marks of distension, and Dr. Guy, that they contain a small quantity of blood, and the medullary substance presents, when sliced, a number of bloody points. With these latter authorities we are inclined to coincide.

When all these signs exist, with the absence of wounds or other injuries which might account for the death, its cause can not be a matter of doubt, but the signs are sometimes wanting, and sometimes their presence admits of another explanation. When syncope occurs at the moment of immersion, we may have no evidence of death by drowning, except the finding of the body in water, or if in falling from a height the head strikes a rock, producing concussion of the brain, or in a state of deep intoxication the subject is accidentally or wilfully thrown into the water. But though the want of any or all the ordinary signs, do not absolutely disprove the theory that drowning was the cause of death, the coincident presence of two of them, even when conjoined with a mortal wound of the head, would, we think, furnish very strong proof that death was caused by drowning, and in the absence of conflicting circumstances, other than the wound, justify a verdict to that effect. These signs are the presence of water in the stomach, identical in composition with that in which the body is found, and the presence of a quantity of froth in the air passages. The value of the first and most constant sign, is much impaired by the difficulty of determining whether the water was introduced before or at the time of death, and also by that of proving its chemical identity with that in which the body is found. The other, however, is very significant, and can be produced only by the simultaneous presence of air and water in the lungs while they are still in motion. The presence of water unmixed with air, would prove that it had flowed in after respiration had ceased. The danger of confounding the expectoration of pneumonia or bronchitis with the froth produced by drowning, is very small, on account of the different characters of the fluids, and we think could almost always be avoided by a careful examination of them. The presence of what any one would regard as a mortal wound, would only serve to throw light upon a case that would otherwise be doubtful, not to disprove positive facts. The subject of the celebrated Crow-bar case, would have died by drowning had he been immersed long enough at any time subsequent to the accident from which he suffered, though he had a wound which no surgeon would have hesitated to pronounce speedily fatal. We therefore think that the presence of froth in the air passages, is a certain sign of death by drowning, subject only to the condition that water was not injected into them before death.

